# Adjunctive use of netarsudil 0.02% in the treatment of refractory glaucoma: a one year analysis

**DOI:** 10.1007/s10792-024-03245-z

**Published:** 2024-07-24

**Authors:** Benjamin Zhou, John Yan, Vladislav P. Bekerman, Albert S. Khouri

**Affiliations:** Rutgers New Jersey Medical School, Institute of Ophthalmology and Visual Science, 90 Bergen St, Newark, NJ 07103 USA

**Keywords:** Netarsudil, Rhopressa, Refractory glaucoma, Glaucoma medications

## Abstract

**Purpose:**

This study evaluates the long-term adjunctive use of netarsudil ophthalmic solution 0.02% in lowering IOP in patients with refractory glaucoma.

**Methods:**

This retrospective chart review study was conducted at a tertiary care center. Patients who were prescribed add-on netarsudil therapy and on ≥ 3 topical glaucoma medications from 01/01/2018 to 08/31/2020 were reviewed. 47 patients (69 eyes) met the inclusion criteria. Baseline IOPs prior to the addition of netarsudil were compared to IOPs measured at 3-, 6-, and 12-month intervals. Any patients with inadequate follow-up or who had glaucoma surgery after netarsudil initiation were excluded.

**Results:**

Median baseline IOP (± SD) was 21 ± 5.8 mmHg (median of 2 visits prior to initiation of netarsudil). At 3-month follow-up, 64 eyes had a median IOP of 16 ± 6.7 mmHg (*p* < 0.01). At 6-month follow-up, 56 eyes had a median IOP of 18 ± 4.6 mmHg (*p* < 0.01). At 12-month follow-up, 44 eyes had a median IOP of 15 ± 6.8 mmHg (*p* < 0.01). At the conclusion of the study, 64% of eyes reached 1 year follow-up due to several reasons.

**Conclusions:**

Patients with refractory glaucoma showed statistically and clinically significant IOP reductions on netarsudil. IOP reduction was stable long-term with the largest decrease in IOP seen at 12 months. Although some patients will still go on to require further laser or incisional surgery, for most patients netarsudil is an effective treatment for adjunctive use in refractory glaucoma.

## Introduction

Glaucoma, an optic neuropathy resulting in visual field loss, is a significant global health problem, affecting nearly 2 million people in the USA and 67 million people worldwide. It is considered the world’s leading cause of irreversible blindness and is estimated to cost the US nearly 1.5 billion annually [1]. Notable risk factors for glaucoma include increased intraocular pressure (IOP), increased age, and African American ethnicity—of which IOP remains the primary modifiable risk factor and main target of medical therapy [2]. Reduction in IOP is the aim of therapy, with greater pre-existing damage and risk factors requiring lower IOP. IOP lowering is achieved through decreasing aqueous production and/or increasing trabecular and uveoscleral outflow [3]. Traditionally, prostaglandin analogues, beta-adrenergic blockers, alpha-adrenergic agonists, and carbonic anhydrase inhibitors have been the mainstay therapy [4].

Netarsudil ophthalmic solution 0.02% is a rho-kinase inhibitor administered into the eye once a day. The eye drop was approved by the FDA in 2017 for the treatment of open angle glaucoma and ocular hypertension [3]. Through the inhibition of rho-associated protein kinase and epinephrine transporter, it exerts its IOP lowering effect by increasing trabecular outflow, decreasing episcleral venous pressure, and possibly decreasing aqueous humor production [3]. It has been demonstrated to be efficacious in lowering IOP as monotherapy and in combination therapy for treatment naïve eyes [5–7]. However, its long-term use in patients with refractory glaucoma has yet to be assessed. This study is an investigation of the long-term efficacy of the adjunctive use of netarsudil in refractory cases of glaucoma requiring three or more topical medications at a tertiary care glaucoma center.

## Methods

This study consists of a retrospective record review of patients with refractory glaucoma on three or more topical medications who received add-on netarsudil 0.02% ophthalmic solution to lower IOP as deemed necessary by the treating physician. Patient records between 01/01/2018 and 08/31/2020 were considered. Baseline patient characteristics were recorded including age, gender, ethnicity, mechanism of glaucoma and prior topical, laser, and surgical treatments.

Inclusion criteria included patients with a confirmed diagnosis of glaucoma (primary or secondary) based on clinical evaluation, structure, and function testing, who were using three or more topical glaucoma agents, and who had two documented IOP measurements prior to initiation of netarsudil adjunctive therapy. In this study, a combination of the following topical glaucoma agents was used by patients prior to initiating netarsudil: beta-blockers, carbonic anhydrase inhibitors, alpha agonists, prostaglandins, and latanoprostene bunod (Vyzulta). Patients were excluded if there was inadequate follow-up after starting netarsudil (did not return for 2–4 months), or if they had any type of glaucoma surgery affecting IOP after starting netarsudil. IOP was recorded at 3-, 6-, and 12-month intervals (windows were ± 4-week at 3 months, ± 2 months at 6 months, and ± 4 months at 12 months). In addition to measuring IOP at each follow-up visit, ocular medications, adverse events, and reasons for discontinuation of netarsudil were recorded as well. If during observation eyes were eliminated from the study, all data after elimination was removed from the analysis. Primary outcomes included absolute and relative IOP reductions from baseline.

A Bonferroni corrected non-parametric Mann–Whitney T-test was used for statistical analysis with statistical significance set at *p* = 0.01. Approval for this study was received from the Institutional Review Board at Rutgers New Jersey Medical School. The study followed the tenets of the Declaration of Helsinki.

## Results

A total of 47 patients (69 eyes) who met the above criteria were identified and included in this analysis. Mean age (± SD) was 72.0 ± 12.2 years. Thirty-six patients (52%) were female and 33 (48%) were male. Demographics were as follow: 15 (32%) patients were Caucasian, 22 (47%) African American, and 10 (21%) Hispanic. Glaucoma diagnoses were as follows: 55 (80%) primary open angle, 4 (6%) uveitic, 4 (6%) neovascular, 2 (3%) normal tension glaucoma, 2 (3%) pseudoexfoliation. Fifty-three (77%) eyes had clinically determined severe glaucoma, 15 (22%) eyes had moderate glaucoma, and one (1%) eye had mild glaucoma according to the Hodapp-Parrish-Anderson Staging System. Prior surgeries included: 21 (30%) seton, 13 (19%) trabeculoplasty, 4 (6%) trabeculectomy, 4 (6%) trabecular bypass iStent, and 1 (1%) iridotomy (Table 1).

**Table 1:** Patient and glaucoma characteristics

Age (mean SD)	72.0 ± 12.2
**Eyes**	69
**Gender**	
Male	33
Female	36
**Glaucoma**	
Primary open angle	55
Pseudoexfoliation	2
Chronic angle closure	2
Uveitic	4
Neovascular	4
Normal tension	2
**Prior laser treatment**	
Trabeculoplasty	13
Iridotomy	1
Prior surgical treatment	
iStent	4
Trabeculectomy	3
Seton	21

Throughout the course of observation, 10 (14%) eyes were eliminated from the study at various time points of follow-up due to subsequent laser or surgical intervention after initiation of netarsudil treatment; 13 (19%) eyes were lost to follow-up and 2 (3%) eyes discontinued therapy due to netarsudil adverse events (both due to conjunctival hyperemia). There were no other adverse events reported. In total 64% were maintained throughout the follow up period.

**Fig. 1:**
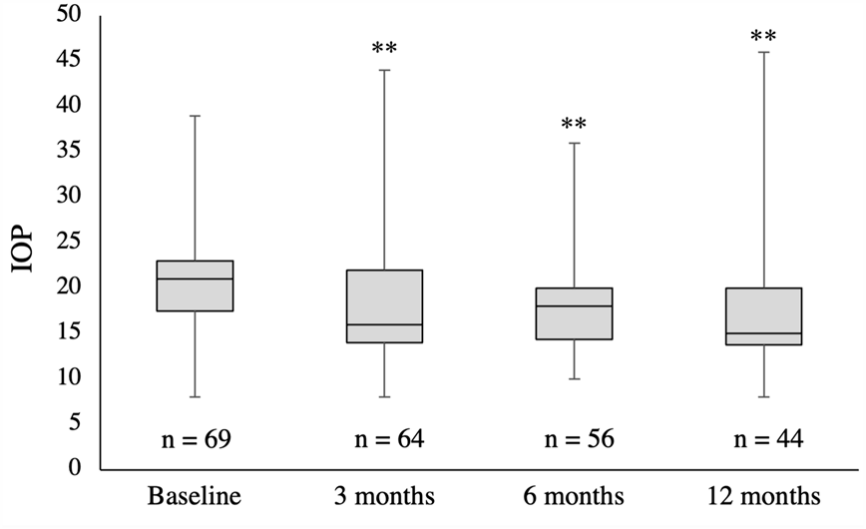
Box plot of intraocular pressures at different time points. Horizontal lines inside the box indicate median. Boxes extend to the 25th and 75th percentile of each group’s distribution of IOP. ** indicates *p* < 0.01 compared to baseline IOP. Legend: IOP = intraocular pressure

**Fig. 2:**
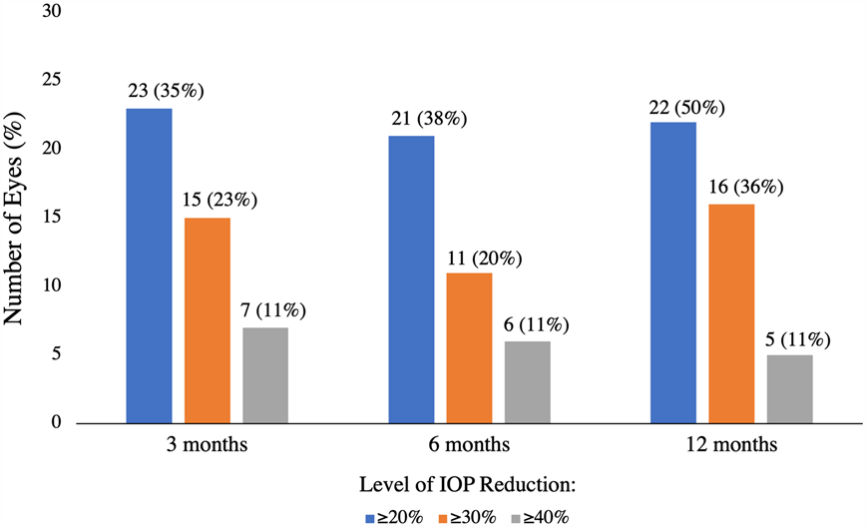
The number and proportion of eyes which showed at least 20%, 30%, and 40% levels of IOP reduction from baseline at corresponding 3-, 6-, and 12-month follow-up intervals

Median IOP (± SD) at baseline was 21 ± 5.8 mmHg (mean of 2 visits prior to initiation of netarsudil). At 3-month follow-up, 64 eyes had a median IOP of 16 ± 6.7 mmHg (*p* < 0.01). At 6-month follow-up, 56 eyes had a median IOP of 18 ± 4.6 mmHg (*p* < 0.01). At 12-month follow-up, 44 eyes had a median IOP of 15 ± 6.8 mmHg (*p* < 0.01) (Fig. 1; Table 2). At 3-months 23 (35%) eyes achieved at least 20% reduction, 15 (23%) eyes achieved at least 30% reduction, and 7 (11%) eyes achieved greater than 40% reduction. At 6-months 21 (38%) eyes achieved at least 20% reduction, 11 (20%) eyes achieved at least 30% reduction, and 6 (11%) eyes achieved greater than 40% reduction. At 12-months 22 (50%) eyes achieved at least 20% reduction, 16 (36%) eyes achieved at least 30% reduction, and 5 (11%) eyes achieved greater than 40% reduction (Fig. 2).

**Table 2:** Intraocular pressure results at baseline and follow-up

	Baseline	3-month	p	6-month	p	12-month	p
Eyes (n)	69	64		56		44	
IOP	20.7 ± 5.8	17.8 ± 6.7	0.00077	17.8 ± 4.6	0.00146	17.4 ± 6.8	0.00074

IOP, Intraocular pressure in mmHg

Of the 10 eyes that were eliminated from the study due to need for subsequent laser or surgical intervention after initiation of netarsudil, 4 eyes underwent glaucoma tube shunts, 3 eyes underwent traditional transscleral cyclophotocoagulation (CPC), 1 eye underwent micropulse cyclophotocoagulation (MPCPC), 1 eye underwent selective laser trabeculoplasty (SLT), and 1 eye underwent MPCPC and SLT. In those eyes, median IOP (± SD) at baseline was 22 ± 5.8, at 1-month was 20 ± 4.9, at 3-months was 16 ± 4.9, at 6-months was 18 ± 3.7, and at 12-months was 18 ± 8.6. Importantly, 7 eyes stayed on netarsudil even after laser or surgical intervention, 2 eyes stopped netarsudil after laser or surgical intervention, and 1 eye initially stopped netarsudil after laser or surgical intervention, and later restarted due to elevated IOP. In addition, the median time to subsequent laser or surgical intervention was 127 days.

## Discussion

This study investigates the long-term efficacy of netarsudil 0.02% when added as an adjunctive therapy in eyes with refractory glaucoma that were on three or more topical medications. Netarsudil has been shown to decrease cellular stiffness, cell adhesion, and actomyosin contraction of the trabecular meshwork and Schlemm’s canal.^9^ While netarsudil was demonstrated to be tolerable and effective in treating glaucoma as monotherapy or in combination therapy for treatment naïve eyes, data concerning its real-world efficacy as an adjunctive agent over longer periods (> 6 months) remains lacking [5, 7, 8].

Our study’s focus on netarsudil’s adjunctive effect is also of particular clinical importance. Despite netarsudil’s approval by the FDA, the assessment of its real-world clinical utility as monotherapy is limited due to the notable cost and formulary barriers. Our study offers insight on netarsudil’s use in patients on multiple medications and in a unique population of patients with refractory glaucoma, as 77% of our cohort was clinically designated as having severe glaucoma.

To the best of our knowledge, this study is the first to follow refractory patients at temporally regimented time points, and the first study to present IOP data beyond 3–6 months. Netarsudil demonstrated significant IOP lowering in our cohort of patients with refractory glaucoma on three or more medications up to 12 months.

Our findings corroborate prior studies which have demonstrated the effectiveness of netarsudil as adjunctive therapy in patients on multiple medications. However, our cohort which represented patients with more severe disease and ethnic diversity had a relatively lower effectiveness in IOP reduction compared to these prior studies [5–8]. Mehta et al. noted an average 3.9 mmHg (17.5%) IOP reduction after mean usage of 54.3 days, while in our study at 3-month follow-up the reduction was only 2.8 mmHg (11%). Another study done by Fridman et al. demonstrated a 3.01 mmHg (16.5%) reduction in patients who had 2 follow-up visits within a 6-month time point [8]. Additionally, the combination therapy arm of the M.O.S.T. trial demonstrated an even higher decrease of 4.32 mmHg (20.5%) at the 3-month mark [5]. Our results at the 12-month mark, which showed a 3.5 mmHg (16%) reduction, are more in-line with the end-point results presented by Mehta et al. and Fridman et al., but still fall short of findings observed in the M.O.S.T trial.

Although our study demonstrated a notably lower IOP reduction compared to these studies after 3 months of treatment it should be restated that our population was comprised of refractory patients who were on multiple pharmacotherapies and with more advanced pathology. We also believe that this differential could be partially due to the elimination of 10 (14%) eyes which required further laser or surgical intervention throughout the course of the study, thus enhancing the influence of netarsudil-respondent eyes. This highlights the fact that although there were overall IOP reductions with netarsurdil in our patient population, there still were patients for whom IOP reductions were not enough for the stage of the disease. These patients required further laser or incisional surgery.

Overall, we suspect that the efficacy of netarsudil as combination therapy across multiple studies may have to do with its unique mechanism of action on the rho kinase pathway which has been shown to relax the trabecular meshwork and enhance conventional aqueous humor drainage and lower episcleral venous pressure [3, 9, 10]. By acting on these previously unaddressed treatment pathways, netarsudil may maintain therapeutic value even in a polypharmaceutical/post-surgical setting.

The only adverse effect requiring discontinuation noted in our study was hyperemia in two eyes (3%). Multiple phase 3 trials have shown hyperemia to be the most common adverse effect, ranging from 47.9 to 60.6%, followed by conjunctival hemorrhage which occurred in 5% to 19% of patients [11–13]. The ROCKET-1 and ROCKET-2 clinical trials reported a 10%-12% discontinuation rate due to adverse events [11]. In our cohort, we attribute the lower discontinuation rate to the likelihood that patients on multiple agents have higher tolerance for adverse effects. It is also likely that eyes on multiple medications had ocular surface disease and conjunctival hyperemia prior to netarsudil therapy. Nonetheless, our results show that the majority of patients tolerated and were able to persist with netarsudil therapy.

Our study is unique in that it provided longer follow-up with 3-, 6-, and 12-month data on efficacy in a refractory glaucoma population. The study population was meant to mirror real-world use of netarsudil. However, the study has several limitations including its retrospective nature, the lack of a comparative control arm, limited sample size, and a heterogenous glaucoma group. Usage of systemic medications (e.g., antihypertensives, vasodilators) were not accounted for. A larger study will be needed to further confirm netarsudil’s efficacy in various types of glaucoma.

## Conclusion

In summary, the results of our study show that adjunctive use of netarsudil in refractory glaucoma patients on three or more medications showed clinically and statistically significant long-term IOP reductions. The data serves to further our understanding of the utility and tolerability of netarsudil in a real-world setting. Further research on the efficacy of netarsudil use in patients with refractory glaucoma is necessary.

## References

[1] Mantravadi AV, Vadhar N (2015) Glaucoma. Prim Care 42(3):437–449. 10.1016/j.pop.2015.05.008 26319348 10.1016/j.pop.2015.05.008

[2] Salowe R, Salinas J, Farbman NH et al (2015) Primary open-angle glaucoma in individuals of African descent: a review of risk factors. J Clin Exp Ophthalmol 6(4):450. 10.4172/2155-9570.1000450 26664770 10.4172/2155-9570.1000450 PMC4671514

[3] Jiang Y, Ondeck C (2020) A review of new medications and future directions of medical therapies in glaucoma. Semin Ophthalmol 35(5–6):280–286. 10.1080/08820538.2020.1818796 33019870 10.1080/08820538.2020.1818796

[4] Jonas JB, Aung T, Bourne RR, Bron AM, Ritch R, Panda-Jonas S (2017) Glaucoma. Lancet 390(10108): 2183–2193. 10.1016/S0140-6736(17)31469-1 10.1016/S0140-6736(17)31469-1 28577860

[5] Zaman F, Gieser SC, Schwartz GF, Swan C, Williams JM (2021) A multicenter, open-label study of netarsudil for the reduction of elevated intraocular pressure in patients with open-angle glaucoma or ocular hypertension in a real-world setting. Curr Med Res Opin 37(6):1011–1020. 10.1080/03007995.2021.1901222 33733980 10.1080/03007995.2021.1901222

[6] Inoue K, Okayama R, Shiokawa M, Ishida K, Tomita G (2018) Efficacy and safety of adding ripasudil to existing treatment regimens for reducing intraocular pressure. Int Ophthalmol 38(1):93–98. 10.1007/s10792-016-0427-9 28063100 10.1007/s10792-016-0427-9

[7] Mehta AA, Kanu LN, Sood-Mendiratta S et al (2021) Experience with netarsudil 0.02% and latanoprostene bunod 0.024% as adjunctive therapy for glaucoma. Eur J Ophthalmol. 10.1177/1120672121998913 33653172 10.1177/1120672121998913

[8] Fridman G, Sadlak N, Eliassi-Rad B, Desai MA (2021) Real-world clinical impact of netarsudil 0.02% at an urban safety-net hospital. J Ocul Pharmacol Ther 37(6):338–342. 10.1089/jop.2020.0112 33983847 10.1089/jop.2020.0112 PMC8328041

[9] Rao PV, Pattabiraman PP, Kopczynski C (2017) Role of the rho GTPase/Rho kinase signaling pathway in pathogenesis and treatment of glaucoma: bench to bedside research. Exp Eye Res 158:23–32. 10.1016/j.exer.2016.08.023 27593914 10.1016/j.exer.2016.08.023 PMC5332476

[10] Yu J, Lan S, Wang R, Maier A, Luan X (2015) Fasudil alleviates traumatic optic neuropathy by inhibiting Rho signaling pathway. Int J Clin Exp Med 8(8):13377–13382 26550269 PMC4612954

[11] Serle JB, Katz LJ, McLaurin E et al (2018) Two phase 3 clinical trials comparing the safety and efficacy of netarsudil to timolol in patients with elevated intraocular pressure: Rho kinase elevated IOP treatment trial 1 and 2 (ROCKET-1 and ROCKET-2). Am J Ophthalmol 186:116–127. 10.1016/j.ajo.2017.11.019 29199013 10.1016/j.ajo.2017.11.019

[12] Khouri AS, Serle JB, Bacharach J et al (2019) Once-daily netarsudil versus twice-daily timolol in patients with elevated intraocular pressure: the randomized phase 3 ROCKET-4 study. Am J Ophthalmol 204:97–104. 10.1016/j.ajo.2019.03.002 30862500 10.1016/j.ajo.2019.03.002

[13] Mehran NA, Sinha S, Razeghinejad R (2020) New glaucoma medications: latanoprostene bunod, netarsudil, and fixed combination netarsudil-latanoprost. Eye (Lond) 34(1):72–88. 10.1038/s41433-019-0671-0 31695162 10.1038/s41433-019-0671-0 PMC7002400

